# 

**Published:** 1879-06

**Authors:** 


PAMPHLETS RECEIVED: Urethrismus, or Chronic Spasmodic Stricture. By F. N. Otis, M.D., Clinical Professor of Genito-urinary Diseases in College of Physicians and Surgeons, New York.

Fourteen Weeks in Botany. By Alphonse Wood, A M. and J. Dorman Steele, Ph.D. (specimen pages). A. S. Barnes & Co , N. Y., 1879.

Excerpta from the Annual Board of Health for 1878. By Joseph Hall, M.D., Sanitary Inspector for the fourth district of New Orleans.

Domestic Sanitation. One of its most important elements as elaborated in a report to the New Orleans Medical and Surgical Association.

Eighteenth Annual Report of the Cincinnati Hospital, for year ending December 31st, 1878. H. M. Jones, Supt.

Transactions of the Detroit Medical and Library Association, April 1879. Published quarterly by the association.

How to elevate the Standard of Medical Education and Medical Teaching. By A. B. Cook, A.M., M.D.

Twelfth Annual Report of the Health Department to the Honorable Common Council of the city of Cincinnati. Thos. C. Minor, M.D., Health officer.

Opium as a Tonic and Alterative, and its Hypodermic use in the Debility and Amaurosis sometimes consequent upon Onanism. By B. A. Pope, M.D. Reprint from February number of *New Orleans Medical and Surgical Journal.*

Response of the Louisville Medical College to charge and specification preferred before the American Medical Association, at the third annual meeting held at Atlanta, Georgia, May 3d, 1879.

On the Permanent Removal of Hair by Electrolysis. By Geo. Henry Fox, M.D., New York.

Ophthalmia Neonatorum. By Richard H. Lewis, M D.

Reprint from *North Carolina Medical Journal*, March, 1879.

How shall the Degree of Doctor of Medicine be conferred. By E. Fletcher Ingalls, M D , Lecturer on Diseases of Chest, etc., in Rush Medical College.

We take pleasure in acknowledging the receipt of a specimen copy of *The Medical Herald of Louisville, Kentucky*, with prospectus. We feel satisfied that the brilliant talent of Dr. R. will add to his galaxy of honors, that of an acceptable and successful journalist. Success to you.

